# Validation of Canadian mothers’ recall of events in labour and delivery with electronic health records

**DOI:** 10.1186/1471-2393-13-S1-S3

**Published:** 2013-01-31

**Authors:** Uilst Bat-Erdene, Amy Metcalfe, Sheila W McDonald, Suzanne C Tough

**Affiliations:** 1 Department of Paediatrics, University of Calgary, Calgary, Alberta, Canada; 2 Department of Community Health Sciences, University of Calgary, Calgary, Alberta, Canada

## Abstract

**Background:**

Maternal report of events that occur during labour and delivery are used extensively in epidemiological research; however, the validity of these data are rarely confirmed. This study aimed to validate maternal self-report of events that occurred in labour and delivery with data found in electronic health records in a Canadian setting**.**

**Methods:**

Data from the All Our Babies study, a prospective community-based cohort of women’s experiences during pregnancy, were linked to electronic health records to assess the validity of maternal recall at four months post-partum of events that occurred during labour and delivery. Sensitivity, specificity and kappa scores were calculated. Results were stratified by maternal age, gravidity and educational attainment.

**Results:**

Maternal recall at four months post-partum was excellent for infant characteristics (gender, birth weight, gestational age, multiple births) and variables related to labour and delivery (mode of delivery, epidural, labour induction) (sensitivity and specificity >85%). Women who had completed a university degree had significantly better recall of labour induction and use of an epidural.

**Conclusion:**

Maternal recall of infant characteristics and events that occurred during labour and delivery is excellent at four months post-partum and is a valid source of information for research purposes.

## Introduction

Maternal report of events that occurred during labour and delivery are used extensively in epidemiological research. Self-report data are commonly collected as they can be obtained efficiently and at less cost than medical chart reviews; furthermore, self-report allows researchers to simultaneously collect other data that may not be available in medical charts or other source documents, such as lifestyle information [1-4]. Although maternally reported data about the events occurring during labour and delivery are widely used, the validity of this data is rarely confirmed. To our knowledge, maternal recall of birth events has not been validated in a Canadian population and few studies have involved electronic health records.

According to previous studies, the validity of maternal recall varies based on the type of information [2-6], the way questions were worded [7], mothers’ socio-economic status [1,8,9], and length of time since the event [9,10]. Other studies, specifically those examining maternal recall of infant birth weight, suggest that age, parity, time since birth and ethnicity do not affect the validity of maternal recall [1,11].

The majority of validation studies have focused on maternal recall of infant birth weight, gestational age and/or mode of delivery [1-6,8-19]. Generally, they have found that maternal recall for these variables is excellent. A US study found that 89% of 46,637 women sampled could recall their infant’s birth weight within one ounce when compared to the weight recorded on the medical charts [13], while a British study found that 91% of 649 mothers were able to recall their infant’s birth weight within 200g compared to medical charts [16]. Another study conducted in the UK, determined that 94.5% of 8037 women could recall their infants gestational age at birth within one week of the gestational age found in the medical charts [8]. Other studies showed that maternal recall of gestational age is less accurate than their recall of birth weight, but concluded that maternal recall of both gestational age and birth weight are valid [1,2,6,9,15,19]. Several studies examining vaginal vs. caesarean delivery also found high agreement (typically >90%) between medical charts and maternal report [5,7,10,12,15,17]. In contrast, conflicting results [5,7,15] have been found for use of forceps during delivery. While one Australian study reported that maternal recall for the use of forceps was 99.7% [7], another Australian study found that only 63.6% of mothers could accurately recall if forceps were used [5].

The aim of this study was to validate maternal self-report at four months post-partum of events that occurred during labour and delivery with data from electronic health records (EHRs) in a Canadian setting. This validation is important because several large birth cohorts [20-22] have been recently developed in Canada, all of which obtained data through questionnaires. Additionally, national surveys conducted by Statistics Canada, such as the Canadian Community Health Survey [23,24] and the Maternity Experience Survey [25,26], also rely on maternal recall of events that occurred during labour and delivery and data from EHRs are not always accessible for research purposes due to cost and time restraints that may prohibit access.

## Methods

Self-reported data on birth outcomes was obtained from the All Our Babies (AOB) study, a prospective community-based cohort of women’s experiences during pregnancy and the post-partum period. Pregnant women were recruited through physicians’ practices, laboratory services, and posters in the community, and were eligible to participate if they were at most 24 weeks and 6 days gestation at the time of recruitment, receiving prenatal care in Calgary, and able to complete the questionnaires in English (n=4,003) [27]. Participants were asked to complete three written questionnaires: the first before 24 weeks of gestation, the second between 34 and 36 weeks, and the third at four months post-partum. Data for this study comes from the questionnaire that women completed at four months post-partum. In total, 3,388 women completed at least one questionnaire, with a retention rate of 85%. A more detailed description of the AOB methodology can be found in McDonald et al [27]. Ethical approval for the AOB study was granted by the University of Calgary’s Conjoint Health Research Ethics Board.

Pregnancy and birth outcome data were obtained directly from electronic health records (EHR) for the hospital admission for labour and delivery. EHRs include data on antenatal risk factors and events that occurred during labour and delivery [28]. Deterministic linkage based on maternal personal health number (PHN), name and other unique identification factors were used to link data from the AOB study with the EHRs. A total of 2,859 women were identified in both datasets. We were unable to link approximately 15% of participants due to missing or erroneous personal health numbers (primary reason), missing postpartum questionnaire data, or delivery outside of hospital without a registered midwife.

Data elements from the AOB questionnaires and the EHR were reviewed, and eight common variables (caesarean delivery, epidural usage, gestational age, infant birth weight, infant gender, labour induction, multiple gestation pregnancy, preterm birth) were identified. Gestational age and birth weight were examined as both continuous and categorical variables. Gestational age was categorized as preterm (<37 weeks), term (37-40 weeks) and post-term (≥41 weeks) [29]. Low birth weight was defined as birth weight less than 2500g.

Validation of the maternal self-report with the EHR was measured by calculating the sensitivity and specificity for measuring bias, and kappa score for measuring precision [18]. Kappa coefficients were considered to represent excellent agreement if the value was greater than 0.75, moderate agreement if the value was between 0.40 to 0.75 and poor agreement if the value was less than 0.40 [12]. The validity of maternal recall for continuous data elements was determined by calculating the proportion of mothers who reported the gestational age and birth weight within one week and 50g increments.

To determine if socio-demographic factors influenced maternal recall, a series of stratified analyses were also conducted by highest level of education (university vs. high school), gravidity (primigravida vs. multigravida), and maternal age at delivery (<35 vs. ≥35). Data on gravidity and education were obtained from the AOB questionnaire and data on maternal age at delivery was obtained from the EHRs. Sensitivity and specificity were compared and values where the 95% confidence intervals did not overlap were considered to be significantly different at α=0.05 level. Chi square tests were used to examine differences in demographic variables between women whose questionnaire data could and could not be linked to EHRs. All analyses were conducted in Stata SE Version 11.

## Results

Characteristics of participants identified in both the AOB dataset and EHRs are described in Table 1. Most of the women reported completing a university education, being in a relationship, and having a household income of $40,000 Canadian or greater. Data from the Maternity Experience Survey indicated that 75.3% of the mothers nationally, compared to 88.6% of the women in this study, have an annual household income of at least $40,000 Canadian [30]. Additionally, data from Statistics Canada indicate that nationally 61.5% of women have completed a university degree compared to 74.9% seen in this sample [31]. Significant differences were observed among women whose questionnaires could and could not be linked to EHRs. Women whose questionnaire data could not be linked to EHRs were significantly less likely to be employed, married or in a common-law relationship, over age 35 at the time of delivery, to own their own homes, to have delivered their infant in a hospital. They also had lower educational attainment and household incomes. No differences were observed based on country of birth, ethnicity, language spoken at home, and gravidity (results not shown).

**Table 1:** Characteristics of women (N=2,859)

Variable	N (%)
Ethnicity	
Caucasian	2,249 (78.7)
Non-Caucasian	593 (20.7)
Employment status	
Employed	2,660 (93.0)
Non-employed	88 (3.1)
Highest level of education obtained	
Less than high school	87 (3.0)
High school	614 (21.5)
College or university	1,769 (61.9)
Graduate school	372 (13.0)
Home ownership	
Own their home	2,214 (77.4)
Not own their home	630 (22.0)
Income	
Less than $40,000	225 (7.9)
$40,000 to $80,000	597 (20.9)
More than $80,000	1,935 (67.7)
Language spoken at home	
English	2,517 (88.0)
Non-English	328 (11.5)
Marital status	
Single, widowed, divorced	142 (5.0)
Married or common-law	2,701 (94.5)
Mother’s place of birth	
Canada	2,222 (77.7)
Other	624 (21.8)
Number of babies delivered	
One	2,657 (92.9)
More than one	38 (1.3)
Place of delivery	
Hospital	2,627 (91.9)
Other	68 (2.4)

Note: Due to item non-response N does not equal 2,859 for all variables and percentages do not sum to 100%

Table 2 shows the overall agreement between self-reported data from the AOB cohort and information from the EHR. Maternal recall at four months post-partum was found to be valid with high sensitivity, specificity and kappa scores for all variables. Sensitivity and specificity were greater than 90% for the majority of variables studied. The variables of epidural usage, labour induction, and post-term births had lower sensitivity and specificity than the other variables studied; however, sensitivity and specificity were all greater than 80%.

**Table 2:** Validity of self-reported data compared to electronic health records

Variable description	Prevalence	Sensitivity (95% CI)	Specificity (95% CI)	True Positives N	False Positives N	False Negatives N	True Negatives N	Kappa (95% CI)
Caesarean delivery	756/2695 (28.1)	99.7 (99.0,100.0)	99.8 (99.6,100.0)	706	3	2	1984	0.99 (0.99,0.99)

Epidural usage	1477/2859 (51.7)	90.7 (89.1,92.2)	82.2 (80.1,84.2)	1340	246	137	1136	0.73 (0.71,0.76)

Infant gender	Males: 1362/2621 (52.0)	99.4 (98.8,99.7)	99.0 (98.2,99.5)	1274	12	8	1180	0.98 (0.98,0.99)

Low birth weight	159/2552 (6.2)	96.5 (92.1,98.9)	97.8 (97.1,98.3)	139	53	5	2355	0.82 (0.77,0.86)

Labour induction	755/2796 (27.0)	87.3 (84.7,89.6)	90.6 (89.2,81.8)	659	192	96	1849	0.75 (0.72,0.78)

Multiple gestation pregnancy	36/2693 (1.3)	94.3 (80.8,99.3)	99.8 (99.6,99.9)	33	5	2	2653	0.90 (0.83,0.97)

Post-term births	1373/2677 (51.3)	94.9 (91.5,97.3)	88.5 (87.1,89.7)	243	279	13	2142	0.57 (0.53,0.61)

Preterm births	208/2677 (7.8)	94.8 (90.6,97.5)	98.4 (97.8,98.8)	182	40	10	2445	0.87 (0.83,0.91)

Note: Prevalence data were obtained from the electronic health records. Denominators differ due to missing data in the EHR.

When examined as continuous variables, the majority of women could accurately recall their infant’s gestational age and birth weight within a margin of error, although exact recall was low. Out of 2,677 mothers, 71.5% of the mothers recalled the exact gestational age of their infant, while 98.3% of the mothers remembered the gestational age of their infant within two weeks (Table 3). At four months post-partum, the exact birth weight of the infant was recalled by 11.6% of 2,552 mothers, and 91.7% of the mothers remembered the birth weight of their infant within 200g (Table 4). The inability of mothers to recall exact gestational age and birth weight has implications when calculating rates of common perinatal outcomes. The prevalence of low birth weight and post-term birth was significantly higher in the AOB data than in the EHRs (Table 5).

**Table 3:** Maternal recall of birth weight (n=2,552)

Difference from exact birth weight (g)	Maternal recall	
	
	n	Percentage (%)
0.00	284	11.13
±50.00	2256	88.40
±100.00	2341	91.73
±150.00	2391	93.69
±200.00	2413	94.55

**Table 4:** Maternal recall of gestational age (n=2,677)

Difference from exact gestational age (weeks)	Maternal recall	
	
	n	Percentage (%)
0	1913	71.46
±1	2570	96.00
±2	2631	98.28

**Table 5:** Impact of maternal recall of continuous variable on categorical outcomes

Variable	All Our Babies Prevalence (95% CI)	Electronic Health Record Prevalence (95% CI)
Low-birth weight	**7.5 (6.5,8.5)**	**5.6 (4.7,6.4)**
Post-term delivery	**19.5 (18.0,21.0)**	**9.4 (8.3,10.4)**
Preterm delivery	8.3 (7.2,9.3)	7.3 (6.3,8.2)

Note: Bold values indicate significance at α<0.05

Stratified analysis revealed that women who had completed a university degree had significantly greater recall of whether their labour was induced (sensitivity=90.1, 95% CI: 87.3-92.4) and epidural usage (sensitivity=92.3, 95% CI: 90.6-93.8) compared to women who had only completed high school or less (labour induction: sensitivity=79.3, 95% CI: 72.8-84.8; epidural: sensitivity=86.6, 95% CI: 82.8-89.7) (Table 6). However, no significant difference was found in any of the outcomes studied by maternal age or gravidity (results not shown).

**Table 6:** Agreements between maternal self-report and the EHR by mother’s highest level of education (n=2,844)

Variable description	Sensitivity (%),(95% CI)		Specificity (%),(95% CI)	
	Highest level of education obtained		Highest level of education obtained	
	University	High school	University	High school
Caesarean delivery	99.6 (98.7,100)	100 (97.7,100)	99.8 (99.4,100)	100 (99.2,100)
Epidural usage	**92.3 (90.6,93.8)**	**86.6 (82.8,89.7)**	82.5 (80.1,84.7)	81.3 (76.5,85.6)
Infant birth weight	99.0 (94.7,100)	89.7 (75.8,97.1)	98.3 (97.6,98.9)	96.0 (94.0,97.5)
Infant gender	99.4 (98.7,99.8)	99.3 (97.5,99.9)	98.8 (97.8,99.4)	99.6 (98.1,100)
Labour induction	**90.1 (87.3,92.4)**	**79.3 (72.8,84.8)**	91.2 (89.7,92.6)	89.0 (85.9,91.6)
Multiple gestation pregnancy	93.3 (77.9,99.2)	100 (39.8,100)	99.9 (99.6,100)	99.7 (98.8,100)
Post-term births	96.0 (92.3,98.3)	90.9 (80.0,97.0)	88.7 (87.1,90.1)	87.6 (84.6,90.2)
Preterm births	95.3 (90.6,98.1)	92.3 (79.1,98.4)	98.9 (98.3,99.3)	96.7 (94.9,98.0)

Note: Bold values indicate significance at α<0.05

## Conclusions

By comparing the self-reported data from women who participated in the AOB study and electronic health records, it was found that maternal self-report of events occurring around the time of labour and delivery are highly valid when recalled four months after delivery. Educational attainment was the only socio-demographic factor that influenced the accuracy of maternal recall in this study. Although women who had completed university had more accurate recall of labour induction and epidural usage than the mothers who had completed high school, it is worth mentioning that both groups of mothers had high rates of recall. It is plausible that women with higher educational attainment asked more questions during labour and delivery and as such were more aware of events during labour and delivery. The literature is conflicting as to whether women with more education have more accurate recall with some studies showing that more educated women had more accurate recall [2,5,19], and other studies showing no effect of education on validity [9,15-17].

A significant association between validity of recall and maternal age and/or gravidity was not observed in this study. Similarly, other studies have not found an association between maternal age and recall [9,16,17,19]. While no studies could be found that assessed the relationship between gravidity and recall, several studies have examined the relationship between parity and maternal recall. Results are conflicting with some studies showing that parity is negatively associated with recall [1,2] while others show that there is no association with parity [3].

In the present study, mothers were able to more accurately report events if they were presented with a list of options (i.e. How was your new baby delivered? A) Vaginally, B) You went into labour but had an emergency caesarean section, C) You did not go into labour and had an emergency caesarean section, D) You had a planned caesarean section) compared to an open or free-text field (i.e. What was your baby’s birth weight?). This difference could also be due to the nature of the question asked. Further work that asks participants the same questions with both categorical and open response options is warranted as this may have important implications for researchers as they strive to reduce participant burden and achieve accurate responses.

Despite the large population, this study is not without limitations. As the present analysis was limited to variables that were captured in both datasets, the validity of socio-demographic and lifestyle factors, mental health, and pregnancy complications could not be assessed. Future studies should assess the validity of these and other important perinatal variables. Some stratification factors (gravidity and education) were self-reported and we cannot exclude the possibility of inaccurate reporting. However, significant differences were not observed with gravidity, and due to social desirability, we presume that women would be more likely to report a higher level of educational attainment that would minimize the difference in reporting seen between groups. Finally, this study was limited to women who could complete a written questionnaire in English. This prohibited an analysis of the impact of English fluency on the validity of maternal recall.

In conclusion, maternal recall at four months post-partum of important events that occurred during labour and delivery is excellent. This study fills a gap in literature and shows that self-reported data from Canadian mothers are valid sources of information with comparison to EHR for research purposes.

## List of Abbreviations

AOB: All Our Babies; CI: Confidence Interval; EHR: Electronic Health Record

## Competing interests

None of the authors have any competing interests to declare

## Author contributions

SCT designed and secured funding for the overall study. AM and SWM designed the sub-study. UBE and AM conducted the analysis. UBE drafted the manuscript. All authors critically reviewed the manuscript and approved the final version for submission.

## Declarations

This article has been published as part of *BMC Pregnancy and Childbirth* Volume 13 Supplement 1, 2013: Preterm Birth: Interdisciplinary Research from the Preterm Birth and Healthy Outcomes Team (PreHOT). The full contents of the supplement are available online at http://www.biomedcentral.com/bmcpregnancychildbirth/supplements/13/S1.

All of the publication fees will be funded by the Preterm Birth and Healthy Outcomes Team Interdisciplinary Team Grant (#200700595) from Alberta Innovates - Health Solutions, formerly the Alberta Heritage Foundation for Medical Research.
